# The bacterial division protein MinDE has an independent function in flagellation

**DOI:** 10.1016/j.jbc.2024.107117

**Published:** 2024-02-23

**Authors:** Pinkilata Pradhan, Ashoka Chary Taviti, Tushar Kant Beuria

**Affiliations:** 1 Infectious Disease Biology, Institute of Life Sciences, Bhubaneswar, Odisha, India; 2 Regional Centre for Biotechnology, Faridabad, Haryana, India

**Keywords:** min system, motility, cell division, two-component system, flagella

## Abstract

Before preparing for division, bacteria stop their motility. During the exponential growth phase in *Escherichia coli*, when the rate of bacterial division is highest, the expression of flagellar genes is repressed and bacterial adhesion is enhanced. Hence, it is evident that cell division and motility in bacteria are linked; however, the specific molecular mechanism by which these two processes are linked is not known. While observing *E. coli*, we found that compared to the WT, the *E. coli* (Δ*min*) cells show higher motility and flagellation. We demonstrated that the higher motility was due to the absence of the Min system and can be restored to normal in the presence of Min proteins, where Min system negatively regulates flagella formation. The Min system in *E. coli* is widely studied for its role in the inhibition of polar Z-ring formation through its pole-to-pole oscillation. However, its role in bacterial motility is not explored. MinD homologs, FlhG and FleN, are known to control flagellar expression through their interaction with FlrA and FleQ, respectively. AtoC, a part of the two-component system AtoSC complex, is homologous to FlrA/FleQ, and the complex is involved in *E. coli* flagellation *via* its interaction with the *fliA* promoter. We have shown that MinD interacts directly with the AtoS of AtoSC complex and controls the *fliA* expression. Our findings suggest that the Min system acts as a link between cell division and motility in *E. coli*.

During bacterial division, a division ring (Z-ring) is formed at the midcell by the divisome complex. FtsZ, a bacterial cytoskeletal protein, assembles in a GTP-dependent manner to form protofilaments, which form the skeleton of the Z-ring (1). The inhibition of FtsZ functions by small molecules leading to inhibition of Z-ring formation and results in cell filamentation (2, 3, 4, 5). Bacteria that divide by binary fission, such as *Escherichia coli*, typically designate their cell division sites at the midpoint. This ensures that cell division should be symmetric, and the resulting daughter cells are approximately uniform in both size and shape (6). The placement of the Z-ring at the midcell in *E. coli* is guided by the Min system through its polar oscillation (7, 8). The Min system in *E. coli* comprises of three proteins named MinC, MinD, and MinE. MinC is an inhibitor of FtsZ assembly; MinD is an ATPase and contains a membrane-targeting sequence that binds to the inner membrane. MinE stimulates MinD ATPase activity, leading to the release of MinD from the membrane. The polar oscillation of the Min system results from the intricate interplay between ATP-dependent membrane association, subsequent MinD oligomerization, and MinE-induced local release of MinD from the membrane upon ATP hydrolysis. MinC essentially acts as a "passive rider" on these oscillations. The membrane-bound MinD forms a complex with MinC and inhibits the Z-ring formation at the poles, thereby facilitating the placement of Z-ring at the midcell. Furthermore, this process is enhanced by the interaction of MinD with FtsZ (9). Apart from regulating cytokinesis in *E. coli*, the Min system is also associated with other cellular processes in bacteria, such as chromosome segregation, virulence, and motility (10, 11, 12).

Motility in bacteria is a complex phenomenon mediated by flagella. Flagellation in *E. coli* is regulated by class I, class II, and class III flagellar genes. FlhDC is a class I gene and a master regulator that regulates the expression of the class II flagellar genes. FliA is a flagella-specific sigma factor that positively regulates the expression of all the class III flagellar genes and thus controls flagellation (13). The expression of *fliA* can be regulated at several levels. The presence of an insertion element at upstream of the *flhDC* promoter can increase the *flhDC* expression, which in turn induces *fliA* gene expression and the motility of the bacteria (14). Similarly, the phosphorylated AtoC, a part of the AtoSC two-component system (TCS), interacts with and positively regulates *fliA* promoter expression. The deletion of AtoSC from *E. coli* genome leads to a nonmotile phenotype (15). Further, FliA protein can positively autoregulate itself and FlgM, upon interaction with FliA, can negatively regulate *fliA* expression (13).

The distribution of flagella on the surface of bacteria shows a particular pattern and maintains a specific position and number. One such pattern regulatory protein, FlhG, is involved during flagellation in *Campylobacter jejuni* (*C. jejuni*). Similarly, in *Pseudomonas aeruginosa* deletion of FleN, an ortholog of FlhG, results in hyperflagellation (16). FlhG/FleN regulate flagellation through their interactions with regulatory proteins FlrA/FleQ, respectively (17, 18, 19). However, FlhG/FleN and FlrA/FleQ are absent in *E. coli.* FlhG/FleN are ATPases and homologous to MinD. FlhG is structurally and functionally similar to MinD (20). Like MinD, FlhG forms a homodimer in the presence of ATP, tethers to the membrane with the help of conserved membrane-targeting sequence, and forms foci at both the poles. Recent reports have shown that the deletion of FlhG in *C. jejuni* results in polar minicells formation, mirroring the effects of MinD deletion in *E. coli*, suggesting that FlhG is functionally similar to MinD (21). Interestingly, the Min system is present in *Pseudomonas* and is not reported for its involvement in flagellation or motility (22). Similarly, FleN deletion in *Pseudomonas* leads to higher flagellation but does not form minicells (16). Whereas, studies indicated that the Min system in some bacteria like *Helicobacter pylori* is involved in bacterial motility (18). The role of the Min system in controlling motility in *E. coli* is not known. In the present study, we investigated the effects of the Min system on motility and flagellation in *E. coli*. We also explored if the homologs of FlrA/FleQ are present in *E. coli* and whether these homologs interact with MinD. Our findings showed that the MinDE complex plays a role during *E. coli* flagellation through its interaction with AtoS of the AtoSC complex and thus by controlling *fliA* expression. In this study, we report for the first time the involvement of MinDE during flagellation in *E. coli*.

## Results

### *E. coli* (Δ min) cells showed hypermotility

**Figure 1:**
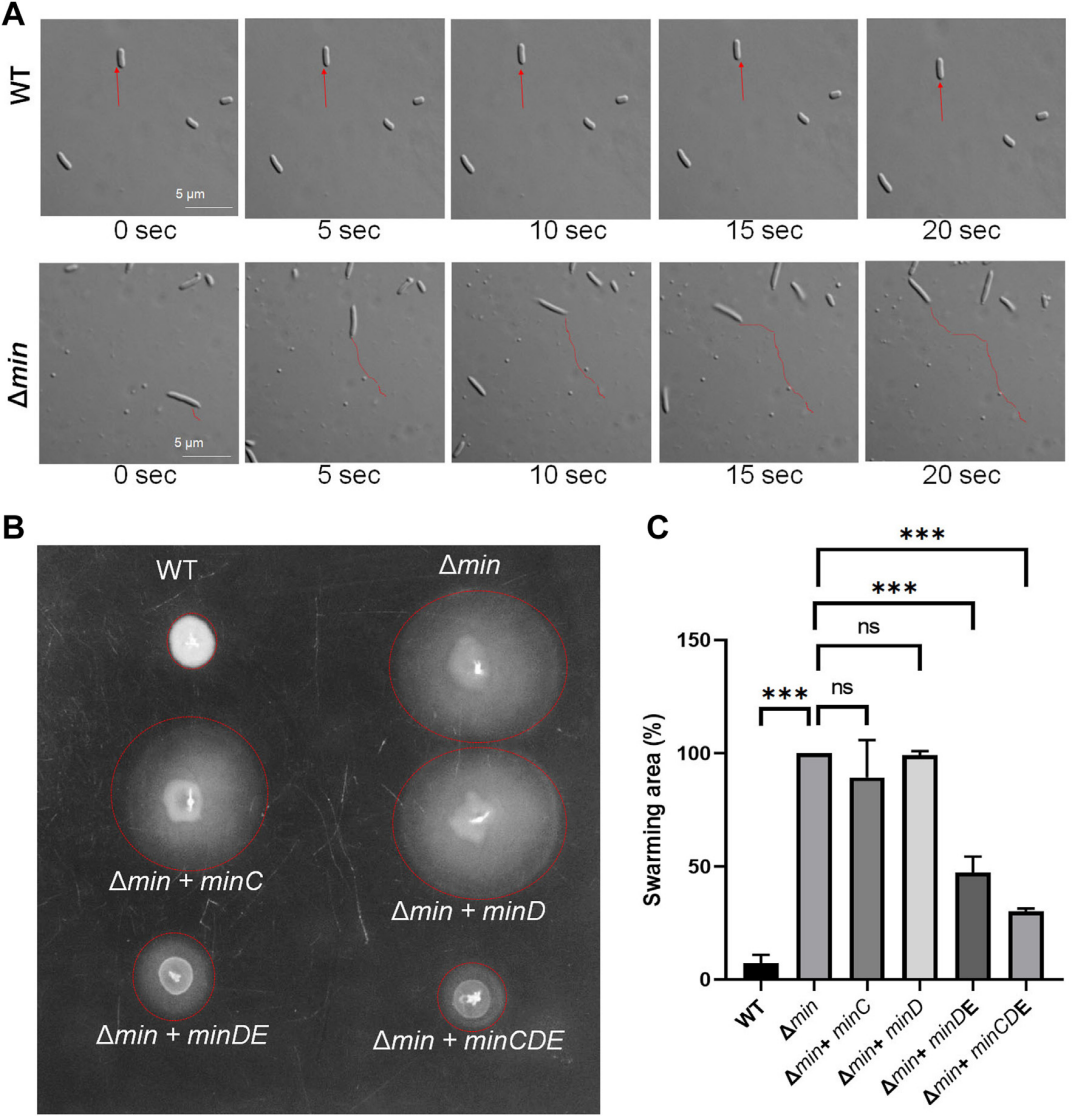
**Min system regulates motility in *Escherichia coli*.***E. coli* MG1655 (WT) WT and *E. coli* JS964 (Δ*min*) were grown in motility media. Few drops were added on to agar over a glass slide and observed under microscope (Olympus BX51). *A*, shows bacterial movement, snapshots of different time points. The *red lines* indicate the path followed by the bacterial during its movement (the scale bar represents 5 μm). *B*, the control strain *E. coli* MG1655 (WT) and *E. coli* (Δ*min*) carrying plasmids (pTrc99a) containing various Min components were grown overnight and 3 μl culture from each strain were spotted on 0.3% soft-agar plates containing ampicillin (100 μg/ml) and 0.1 mM IPTG. The plates were incubated at 37 °C for 12 to 18 h and bacterial motility was observed. *C*, the swarming area percentage in each strain in the form of bar graph. Error bars shows the ± SEM determined and *p* values (<0.05) were determined using an unpaired parametric *t* test (n = 3).

During our observation of *E. coli* (Δ *min*) cells under microscope, we found that compared to *E. coli* WT (MG1655) cells, *E. coli* (Δ *min*) cells showed random and faster movement (Fig. 1*A*). To check whether this motility is due to the absence of the Min system, we examined the motility of *E. coli* (WT), *E. coli* (Δ*min*), and *E. coli* (Δ*min*) complemented with *minCDE* cells using live-cell imaging microscope. We observed that the WT cells showed little movement under this condition, whereas, *E. coli* (Δ*min*) cells showed considerably high motility (Movies S1 and S2). When *E. coli* (Δ*min*) cells were complemented with plasmid containing *minCDE*, the WT phenotype of the cells was regained (Movie S3). To further confirm the motility of the above strains we performed soft agar motility assay, which showed similar results, that is, *E. coli* (WT) showed least motility, *E. coli* (Δ*min*) showed high motility, and *E. coli* (Δ*min*) complemented with *minCDE* showed reduced motility (Fig. 1, *B* and *C*). Our above observations indicated that the Min system might be involved in *E. coli* motility.

### Min system regulates flagellation in *E. coli*

**Figure 2:**
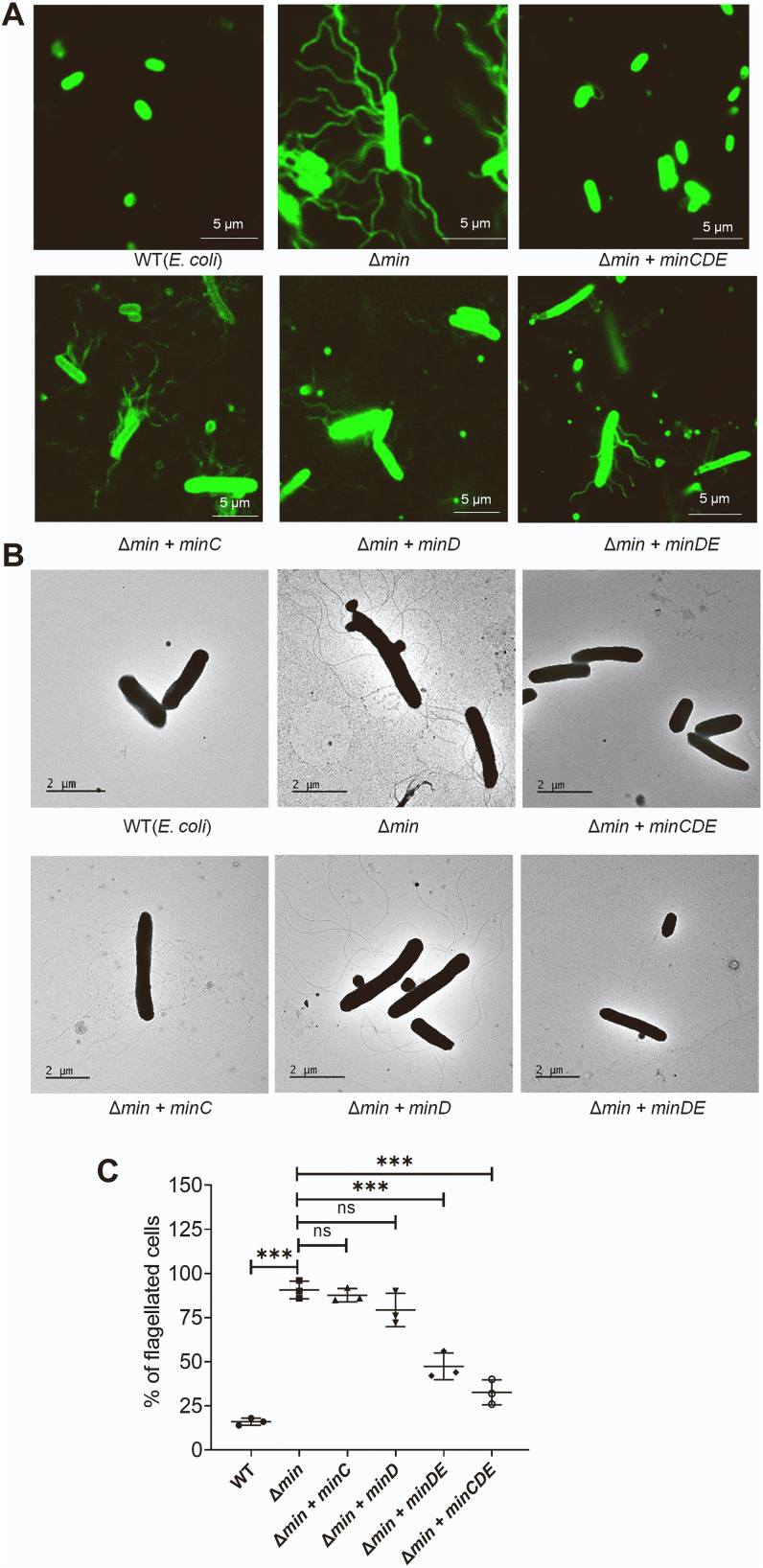
**Phenotype of the**Δ**min cells.** The *Escherichia coli* MG1655 (WT)*, E. coli* (Δ*min*), and *E. coli* (Δ*min*) with various min components complemented cells were grown and bacterial morphology was observed using different techniques. *A*, *E. coli* flagella stained with Alexa Fluro-488 and observed using fluorescence microscope (the scale bar represents 5 μm). *B*, transmission electron micrographs showing the presence of flagella in different bacterial strains (the scale bar represents 2 μm). *C*, the %age of flagellated cells in each observed strain represent as scatter plot. Error bars shows the ± SEM values of three replicates (n = 50) determined using an unpaired parametric *t* test.

Increased flagellation or flagellar activity could cause hypermotility in bacteria (23). So, the observed hypermotility in *E. coli* (Δ*min*) cells could be due to an increase in the flagellation or flagellar activity. To visualize the flagellation, we stained *E. coli* using Alexa Fluor 488, which binds to the amine-rich protein flagellin, and was observed under a fluorescent microscope. Our results showed that *E. coli* (Δ*min*) cells were hyperflagellated that are characterized by dense flagella located all over the cells (Fig. 2*A*). However, we did not observe any flagella in WT cells (Fig. 2*A*). Furthermore, the transmission electron microscopy analysis also confirmed that *E. coli* (Δ*min*) cells contain intact multiple flagella, whereas WT cells are majorly lacking flagella (Fig. 2*B*). Our result was also supported by the previous reports that the lab strain MG1655 (CGSC 6300) shows no or minimal motility in laboratory conditions and thus the flagella could not be visualized in these cells (24).

To identify the components of the Min system that are responsible for the flagellar regulation, we performed motility assay, Alexa Fluor 488 staining and transmission electron microscopy (TEM) analysis of *E. coli* (Δ*min*) cells and *E. coli* (Δ*min*) cells complemented with different Min components (25). Consistent with the previous results, our analysis revealed hypermotility, dense flagellation, and longer flagella in *E. coli* (Δ*min*) cells when compared to WT *E. coli* (MG1655) (Fig. 2). However, when *E. coli* (Δ*min*) cells were complemented with plasmids expressing MinCDE or MinDE proteins, the cells exhibited lower motility than *E. coli* (Δ*min*) cells (Fig. 1, *B* and *C*). Further, we observed flagellation using fluorescence microscopy and TEM imaging. No reduction in the flagellation was observed when *E. coli* (Δ*min*) was complemented with *minC*, or *minD*, or *minE* individually. However, *minDE* or *minCDE* complementation leads to a significant decrease in flagellation (Figs. 2 and S1). It is known that neither MinD nor MinE alone can restore the requirement of the Min system in cell division, and a similar trend was observed in flagellar regulation (26, 27). Further, the number of bacteria containing flagella was determined and plotted (Fig. 2*C*). It was found that ∼90% of the *E. coli* (Δ*min*) cells possess flagella, which decreased significantly (by >50%) when the cells were complemented with *minCDE*.

### Min system negatively regulates the expression of flagellar genes

**Figure 3:**
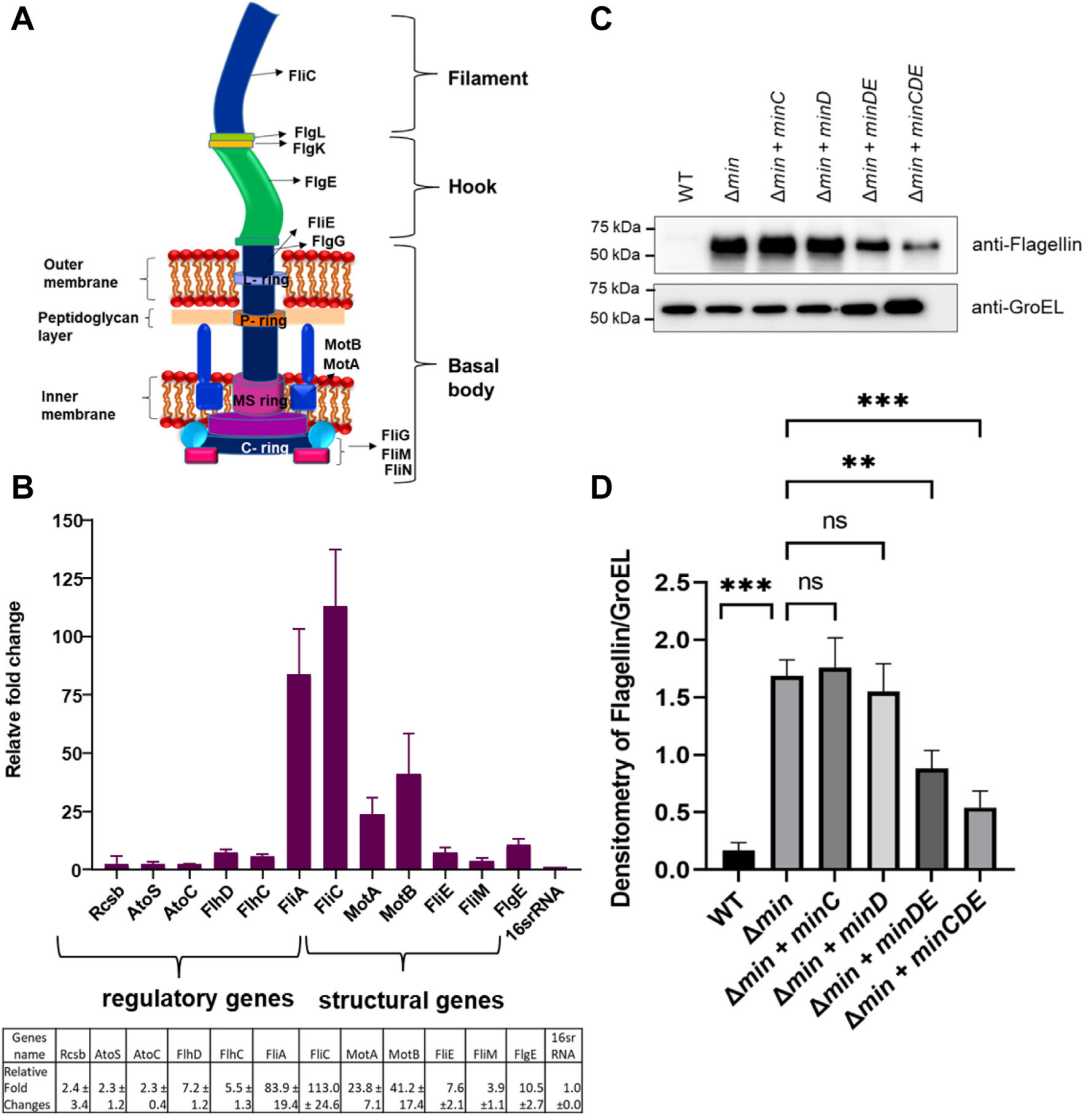
**Flagellar gene expression in*Escherichiacoli* (Δmin) cells.***A*, *cartoon representation* showing the flagella machinery and proteins involved in it. *B*, the RT-PCR analysis of flagellar gene expression in *E. coli* (Δ*min*) cells compared to *E. coli* (WT). Both the types of cells were grown in LB broth, total RNA was isolated, complimentary DNA was synthesized and RT-PCR was performed. The expression of flagellar genes in *E. coli* (Δ*min*) cells was plotted against WT. *C*, the Western blot of flagellin expression in *E. coli* (WT), *E. coli* (Δ*min*), and *E. coli* (Δ*min*) cells complemented with different min components using anti-flagellin antibody was performed. *D*, the relative intensity of flagellin with respect to GroEL was determined and plotted. The error bars show the ± SEM values and *p* values using an unpaired parametric *t* test. Each experiment was repeated three times.

*E. coli* motility relies on flagella rotation, and *E. coli* (Δ*min*) cells show hyperflagellation and increased motility. To understand how the hyperflagellated morphology is happening in *E. coli* (Δ*min*) cells, we checked the expression of flagellar genes and their regulators in the absence of Min system. A flagellum is consisting of several structural proteins, which are governed by its regulators (Fig. 3*A*). The flagellar expression is majorly controlled by the transcription factor FlhDC, a master regulator of flagellar expression (28, 29). FlhDC regulates the expression of the *fliA* gene, which encodes FliA, sigma factor 28 that acts as a regulator for several flagellar genes such as *fliC* (codes for Flagellin), *motA*, and *motB* (30). In order to understand how min proteins might be regulating the expression of flagellar genes and their regulators, we performed quantitative reverse transcription-PCR (qRT-PCR) for flagellar regulators *flhDC, fliA*; structural genes *fliC, flgE*, *fliE*; motor genes *motA*, *motB*, and transcription factor *Rcsb*. Our result showed that compared to the WT *E. coli* the expression of *fliA, motA, motB, and fliC* were significantly upregulated in the *E. coli* (Δ*min*) cells (Fig. 3*B*). Whereas, the expression of *flhDC*, the transcription factor and the global regulator, did not change much in the absence of Min system. To verify this observation, we performed Western blot analysis using anti-flagellin antibody. We examined flagellin (FliC) production in *E. coli* (WT), *E. coli* (Δ*min*), and *E. coli* (Δ*min*) cells complemented with different min components. The *minC*, *minD*, and *minE* complementation did not affect the flagellin production, whereas it decreased considerably when complemented with *minDE* or *minCDE* (Figs. 3, *C* and *D* and S1). A high level of flagellin protein production was found in *E. coli* (Δ*min*) compared to the *E. coli* (WT) cells (Fig. 3, *C* and *D*). Our qRT-PCR findings align with and support this result.

### The role of Min system in controlling *fliA* promoter activity

**Figure 4:**
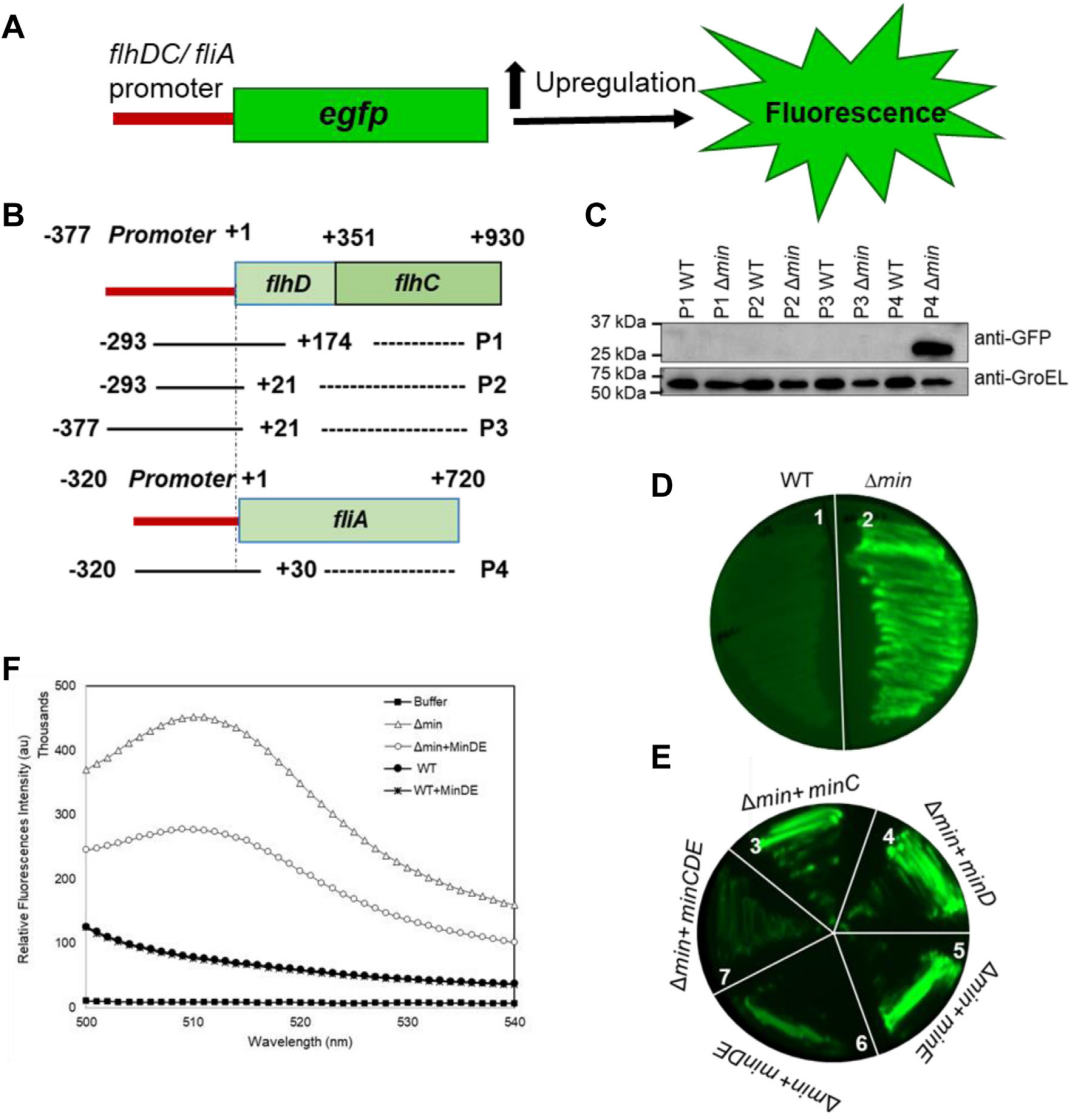
**eGFP reporter assay.** Native promoters of *flhDC* and *fliA* were cloned with eGFP reporter to check the expression of *flhDC* and *fliA* gene. eGFP expression was checked with anti GFP antibody, while keeping anti-GroEL as loading control. *A*, the *pictorial representation* of eGFP reporter assay was shown. *B*, the *schematic representation* of different truncated *flhDC* and *fliA* promoters constructed for the promoter assay. *C*, the expression of eGFP in the presence of different promoter constructs in *E. coli* (Δ*min*) cells and *E. coli* (WT) cells (P1 = *flhDC* 467 bp, P2 = *flhDC* 314 bp, P3 = *flhDC* 398 bp, P4 = *fliA* 350 bp). *D*, shows the *fliA* promoter activity in WT and in Δ*min* system (1): pCDFDuet-1-*fliAp*-eGFP-WT, (2): pCDFDuet-1- *fliAp*-eGFP (Δ*min*). *E*, shows *fliA* promoter with eGFP reporter was cloned into pCDFDuet-1 vector and the expression of *fliA* promoter was studied with various complementation of min proteins (3): pCDFDuet-1-*fliAp*-eGFP/ Δ*min +minC*, (4): pCDFDuet-1- *fliAp*-eGFP/ Δ*min +minD*, (5): pCDFDuet-1- *fliAp*-eGFP/ Δ*min* +*minE*, (6): pCDFDuet-1-*fliAp*-eGFP/ Δ*min +minDE* (7): pCDFDuet-1-*fliAp*-eGFP/ Δ*min +minCDE*. *F*, shows the eGFP fluorescent intensity in *E. coli* (WT), *E. coli* (Δ*min*), and *E. coli* (Δ*min*) cells complemented with *minDE*. eGFP, enhanced green fluorescent protein.

In *E. coli*, FlhDC binds to the promoter region of *fliA* and regulates its expression (14). Thus, the change in either *flhDC* or *fliA* expression can affect the flagellation. In order to understand how the Min system is affecting the *flhDC* or *fliA* expression, we examined the strength of *flhDC* and *fliA* promoter activity in WT and *E. coli* (Δ*min*) cells using an enhanced green fluorescent protein (eGFP) reporter assay (Fig. 4, *A* and *B*). A promoter-less pET22b-eGFP vector was constructed by removing its T7 promoter (Fig. S2) and then the native *E. coli fliA* promoter or the *flhDC* promoter was cloned into it. The promoter activity was studied by measuring the expression of GFP by Western blot using an anti-GFP antibody (Fig. 4*C*). We observed that in *E. coli* (Δ *min*) cells, the *fliA* promoter got activated significantly, whereas, no activity was observed for the *flhDC* promoter. The results suggested that the Min system does not affect the *flhDC* expression, whereas, it negatively affects the *filA* expression. We further found that the complementation of *E. coli* (Δ *min*) cells with *minDE* or *minCDE* lead to reduced eGFP expression, whereas, complementation with any of the single Min components did not affect eGFP expression (Fig. 4, *D* and *E*). The eGFP intensity was also measured using a fluorimeter and we found that compared to the *E. coli* (Δ *min*) cells, the induction of *minDE* reduced the eGFP expression (Fig. 4*F*). Our results showed that the *fliA* promoter activity was low in *E. coli* (WT) cells and high in *E. coli* (Δ *min*) cells. Moreover, the *fliA* promoter activity was reduced when *E. coli* (Δ *min*) cells were complemented with *minDE* indicated the role of MinDE in controlling *fliA* expression.

### MinD does not interact with FlhDC, a class I transcription factor of flagellar regulation

FlhDC, a class-I flagellar protein, controls the expression and regulation of the class-II flagellar protein FliA, which in turn controls the expression of class-III genes (*fliC*, *motAB*, *cheAW*, *flgKL*, and *flgMN*) (31). Our results showed an upregulation of the *fliA* gene and other flagellar genes in *E. coli* (Δ*min*) cells (Fig. 3*B*) and this can be rescued with MinDE complementation. Thus, there is a possibility that MinDE represses the expression of the *fliA* promoter through binding to the FlhDC complex or to the promoter region of *fliA*. Our bioinformatics analysis showed that MinDE lacks DNA-binding domain, suggesting that MinDE does not bind to the promoter region of *fliA*; however, it may bind to the FlhDC complex, a class I transcriptional factor that controls *fliA* expression. In order to check this, we performed yeast two-hybrid assay. The yeast two-hybrid assay showed that MinC, MinD, or MinE did not interact with FlhDC or with other flagellar proteins (FliA or FliC) (Figs. 6*A* and S3). Our findings suggested that the regulation of flagellar expression by MinDE is not through its interaction with flagellar master regulators and maybe through some other mechanism.

### MinD homologs are flagella regulatory proteins

**Figure 5:**
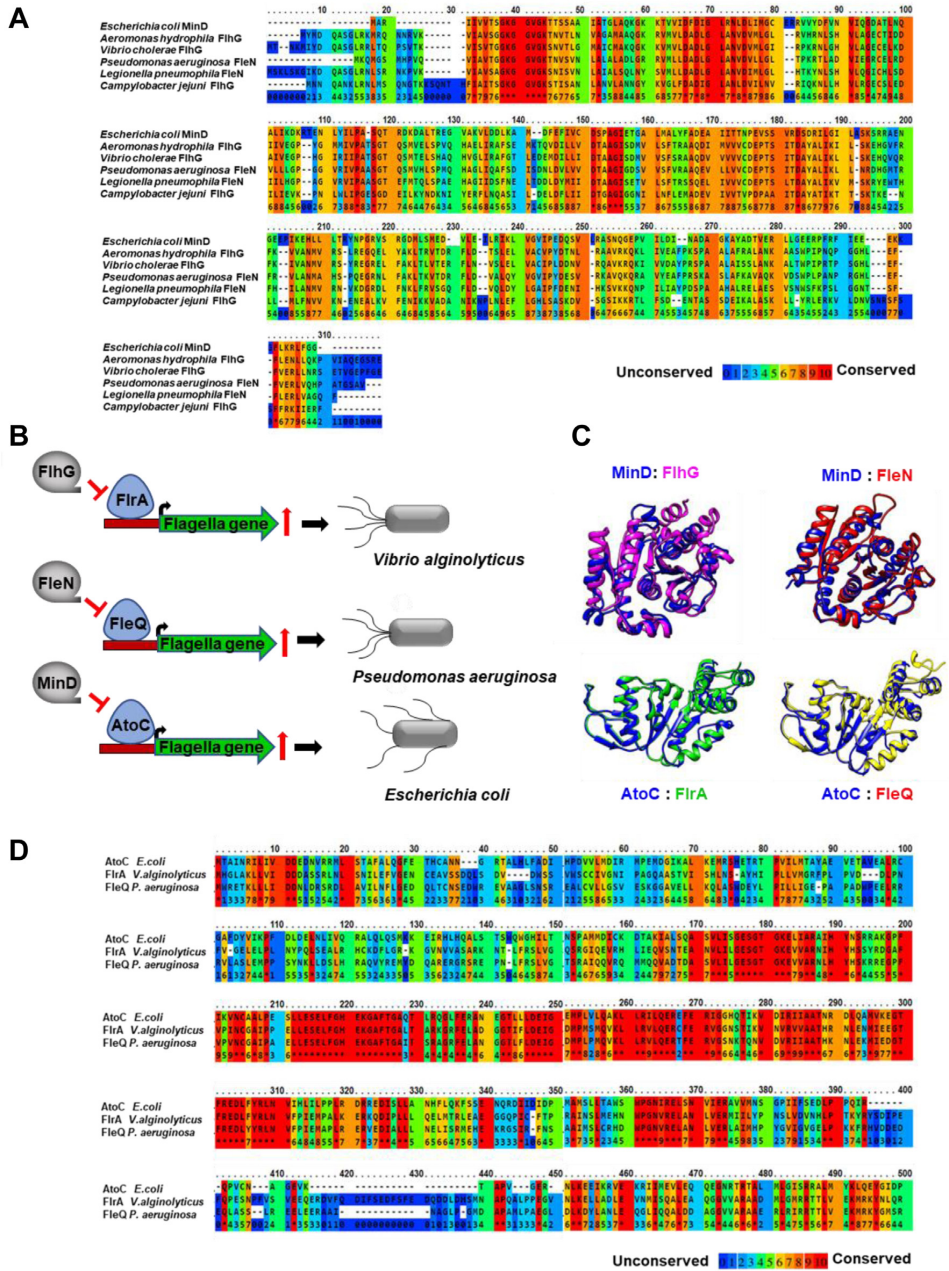
***Escherichia coli* MinD homologs in bacteria and MinD binding partners for flagella synthesis.** Multiple sequence alignment of MinD homologs from different bacteria was performed using Praline multiple sequence alignment program and is shown in *A*. *B*, *schematic diagram* for MinD homologs and their regulatory partners involved in flagellation in different bacteria. *C*, shows the structural alignment of MinD (PDB: 3Q9L), FlhG (PDB: 4RZ2), and FleN (PDB: 5J1J) was performed using Chimera (MinD-*blue colour ribbon*, FlhG-*pink colour ribbon*, and FleN-*red colour ribbon*). This panel also shows the structural alignment of “FleQ (139aa–394aa) with “AtoC” (139aa–378aa) and “FlrA” (143aa–374aa) with “AtoC” (139aa–378aa). FleQ, FlrA, and AtoC structure was downloaded from Alpha fold database and the structure was analyzed by PROCHEK. The structural alignment of FleQ (*Pseudomonas aeruginosa*), FlrA (*Vibrio alginolyticus*), and AtoC (*Escherichia coli*) performed using Chimera (AtoC-*blue*/FlrA-*green*/FleQ-*yellow*). *D*, shows the multiple sequence alignment of FleQ, FlrA, and AtoC.

As MinDE did not directly interact with FlhDC, it encouraged us to identify the partners of MinDE that are involved in flagellar regulation. To understand the roles of MinDE homologs in other bacteria, we performed a BLAST search. Interestingly, our result showed that MinD homologs such as FlhG/FleN are flagella regulatory proteins (16, 32, 33). Multiple sequence alignments of MinD homologs showed highly conserved regions between these proteins (Fig. 5*A*). FlhG is a MinD-like ATPase that binds to the cell membrane and controls the flagella number in *Vibrio alginolyticus* (20). Similarly, FleN, another homolog of MinD, controls the flagella number in *P. aeruginosa* (16). We further performed structural alignment between MinD and FlhG/FleN. The results showed that the structure of MinD is quite similar to that of FlhG (RMSD = 1.05 Å) and FleN (RMSD = 1.09 Å) (Fig. 5*C*). Interestingly, both FlhG and FleN are absent in *E. coli.* As in *E. coli*, MinD is a part of the Min system; these observations indicated a possible involvement of the Min system during the flagellation in *E. coli*.

### MinD interacts with AtoSC, a homolog of FlrA/FleQ

Like MinD, its homologs FlhG/FleN also do not contain any DNA-binding domains, whereas they negatively regulate flagellar gene expression by interacting with sigma-54–dependent regulators FlrA/FleQ, respectively (Fig. 5*B*). FlrA and FleQ interact with the promoter region of the *fliA* gene and induce its expression, which then positively regulates the expression of flagellar genes (34, 35, 36). We hypothesized that MinD might work in a similar manner by interacting with a regulatory protein that is similar to FlrA/FleQ (Fig. 5*B*). To identify their homolog in *E. coli*, we performed a BLAST search of FlrA/FleQ against the *E. coli* K12 genome. Interestingly, we found that the *E. coli* protein AtoC showed more than 40% similarities with these proteins, and upon multiple sequence alignments, we found that these proteins share several conserved regions (Fig. 5*D*). Further, the structural alignment showed that AtoC possesses similar folds as both the proteins, with RMSD 0.5-0.8 Å (Fig. 5*C*). We hypothesized that MinD may directly interact with AtoC and in turn, regulates the flagellar gene expression in *E. coli*.

**Figure 6:**
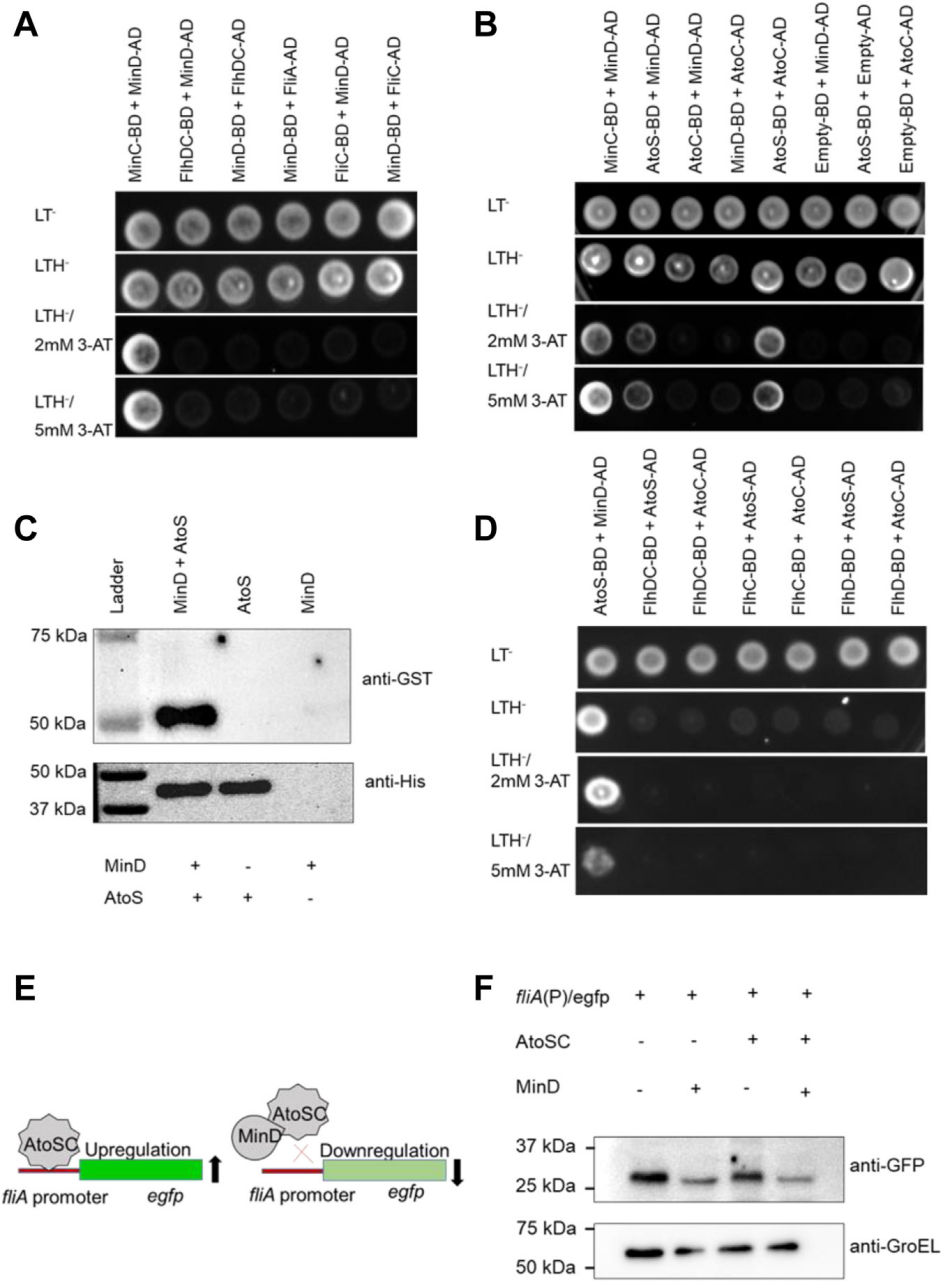
**Interaction of MinD with AtoSC, FlhDC, and role in *fliA* expression.** Interaction between different proteins was assessed through HIS3 reporter assay using yeast two-hybrid system. Various combinations of proteins were cotransformed into HFY7c yeast strain, and 10 μl of each cotransformed cells were spotted on synthetic defined (SD) medium as described in methods section. The cells were grown at 30 °C for 3 to 4 days. Positive interaction was indicated by the presence of colonies in -Leu-Trp-His plates with 3-AT. *A*, shows the interaction of MinD with FlhDC, FliA, and FliC flagellar proteins. *B*, shows the interaction of MinD with AtoC and AtoS on yeast two-hybrid assay. Further, to show the interaction between MinD and AtoS in *in vitro* system, a pull-down assay was performed using His-AtoS and GST-MinD purified proteins. His-AtoS was pulled using Cobalt NTA resin and a Western blot was performed using anti-His or anti-GST antibodies. The Western blot is shown in *C*. The interaction of AtoSC with FlhDC through yeast two-hybrid system is shown on *D*. *E*, shows the schematic representation of eGFP reporter assay. *F*, to check the role of MinD and AtoSC in regulating expression of *fliA* promoter, eGFP expression was monitored with an anti-GFP antibody. The plasmid containing MinD and cyto-AtoSC was transferred into pET-P4-eGFP/C41strain the expression of eGFP was monitored using Western blot. 3-AT, 3-amino-1,2,4-triazole; eGFP, enhanced green fluorescent protein.

To validate our hypothesis, we performed yeast two-hybrid assay, which showed no direct interaction between MinD and AtoC (Fig. 6*B*). AtoC is generally present as AtoSC complex, where AtoS is the sensor histidine kinase that regulates AtoC functions (37). A study by Theodorou *et al.* showed that AtoSC is a TCS that positively regulates motility and flagellar gene expression in *E. coli* (15). Thus, we were curious to know if MinD interacts with the AtoSC complex and in turn controls motility. Interestingly, our yeast two-hybrid result showed that MinD, in fact, directly interacts with AtoS rather than AtoC (Fig. 6*B*). To further validate the MinD and AtoS interaction, we performed pull-down assay. For this, we cloned and purified GST-MinD and His-tagged cytoplasmic parts of AtoS (232–608aa, cyto-AtoS). GST-MinD (5 μM) was incubated with His-cyto-AtoS (5 μM) and pulled down using cobalt resin. MinD that bound with cyto-AtoS was eluted using imidazole, and Western blotting was performed using anti-GST and anti-His antibodies. The pull-down assay showed that MinD directly interacts with cyto-AtoS (Fig. 6*C*). Additionally, we examined the interaction of AtoSC with flagella regulator FlhDC using a yeast two-hybrid assay, which did not show any interaction (Fig. 6*D*). Our result was consistent with the previous findings that AtoC is probably interacting with the promoter region of *fliA* and *flhDC* operons (15).

### AtoSC regulates *fliA* expression through its interaction with *fliA* promoter, whereas MinD regulates flagellation through its interaction with AtoS of AtoSC

It has previously been established that the AtoSC complex positively regulates the motility in *E. coli* (15). As MinD directly interacts with AtoS, we wanted to determine the impact of the MinD–AtoS interaction on bacterial motility. We performed an eGFP reporter assay to study the effect of MinD and AtoSC on *fliA* promoter activity (Fig. 6*E*). We transferred cyto-AtoSC and MinD into pET-P4-eGFP/C41 cells and found that *fliA* promoter activity was high in the presence of AtoSC, whereas the promoter activity went down when MinD was expressed (Fig. 6*F*). The results suggested that MinD interacts with AtoS to control the flagellation in *E. coli*.

### MinDE complex regulates AtoSC phosphorylation

**Figure 7:**
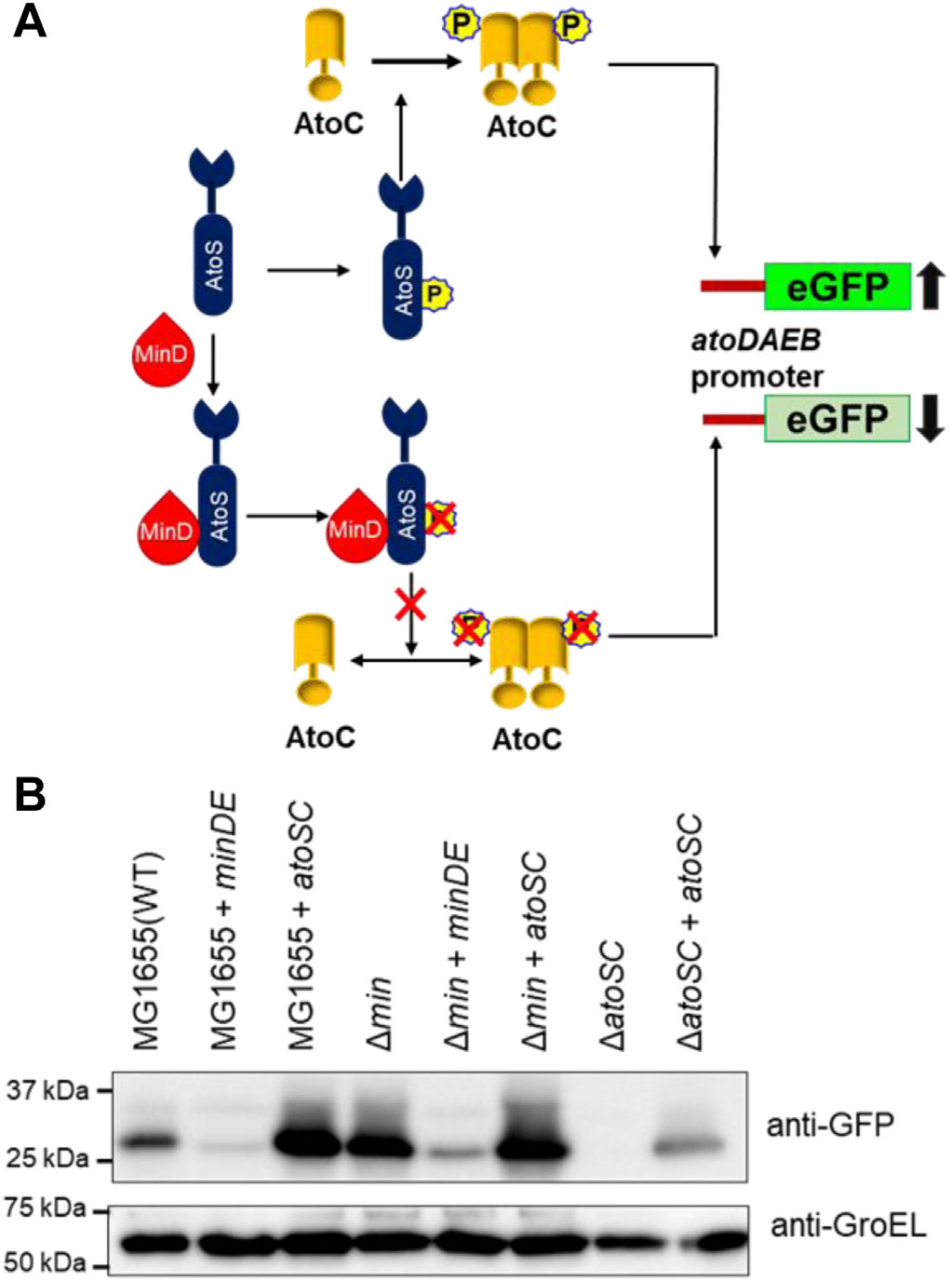
**Effect of MinDE on AtoSC function.***A*, a *pictorial representation* of the pathway of AtoSC mediated *atoDAEB* gene expression was shown. *B*, shows the effect of MinDE and AtoSC on *atoDAEB* promoter activity. The pACYCDuet-1- *tac* -*minDE*, and pACYCDuet-1- *tac*-*atoSC* plasmids along with pET22b-*atoDAEBp*-eGFP vector transferred into the *E. coli* (WT), *E. coli* (Δ*min*), and *E. coli* (Δ*atoSC*) cells and the *atoDAEB* promoter activity was studied using anti-GFP antibody while GroEL was used as loading control. eGFP, enhanced green fluorescent protein.

Theodorou *et al.* have shown that phosphorylation of AtoC is necessary for regulating motility. So, next we wanted to measure the phosphorylation status of AtoSC in the presence of MinDE, but we failed to measure it directly (data not shown). Upon stimuli, AtoS undergoes autophosphorylation and transfers its phosphate group to AtoC (37). The phosphorylated AtoC then binds to the *atoDAEB* promoter and positively regulates its expression (Fig. 7*A*). It was also shown that the ATP-binding–deficient and phosphorylation-deficient mutants of AtoS were unable to activate *atoDAEB* promoter activity. In order to understand the effect of MinD on regulating AtoS phosphorylation, we constructed a reporter assay where the *atoDAEB* promoter was cloned with a GFP reporter and the promoter activity was studied in the presence of AtoSC and MinDE. A positive regulation of AtoS phosphorylation will lead to higher AtoC phosphorylation and activation of the *atoDAEB* promoter, leading to higher GFP expression. Our results showed that in the presence of AtoSC, the promoter activity of the *atoDAEB* is enhanced; however, the expression of MinDE in these cells reduced the *atoDAEB* promoter activity, indicating the involvement of MinDE in negatively regulating AtoS phosphorylation (Fig. 7*B*). It is reported that phosphorylation of AtoC leads to higher flagellation and motility in *E. coli*. As MinDE also negatively regulates flagellation (Fig. 2), the results indicate that inhibition of flagellation in *E. coli* may be attributed to MinDE-mediated inhibition of phosphorylation of the AtoSC complex.

## Discussion

Simultaneous examination of bacterial division and motility is extremely challenging; thus the relationship between cell division and cell motility is poorly understood. A recent study showed that *Myxococcus xanthus* stops its motility during its division and resumes only after completion of the division (38). Similarly, during the exponential growth of *E. coli*, the flagellar gene expression is repressed and bacterial adhesion is enhanced (39). Likewise, in *Caulobacter crescentus*, before undergoing division, the bacterium modifies its flagella into a stalk-like structure that helps it to attach to the surface and stop its motility (40). Thus, it is evident that there is a direct link between cell division and motility in bacteria; however, the specific molecular mechanism by which these two processes are linked is not yet clear. While observing the *E. coli* under live-cell microscopy, we found that compared to the *E. coli* (WT), the *E. coli* (Δ *min*) bacteria show higher motility (Fig. 1). We further confirmed using the soft agar motility assay that the hypermotility was due to the absence of Min proteins and can be rescued by overexpressing Min proteins (Fig. 1). Specifically, MinDE and MinCDE complementation was able to rescue the motility phenotype considerably, whereas individual min proteins could not. Subsequently, we noted that the increased motility was attributed to hyperflagellation in the *E. coli* (Δ *min*) cells (Fig. 2). The Min system in *E. coli* is widely studied for its role in inhibiting polar Z-ring formation and guiding proper cell division. However, its role in *E. coli* motility was never explored. In this study, we focused on understanding the role of the Min system in bacterial motility.

The motility of various bacteria differs from each other and is dependent on the environment. Although the basic design of the bacterial locomotory organ, the flagellum, is similar in all species, there are differences in the number and arrangement of the flagella. In addition, the mechanisms of flagellar biosynthesis and flagellar regulatory machinery differ between species. Flagellation in different bacteria is controlled by >60 genes that are majorly divided into class I, class II, and class III flagellar genes. The class I flagellar genes act as the master regulator, which directly regulates the promoter activity of class II flagellar genes and controls the expression of class III flagellar genes. FlhDC, FleQ, and FlrA are the most common class I master regulators found in different flagellated bacteria (41, 42, 43). The class-II operon encodes for the genes responsible for basal body formation (*i.e.*, FliM, FliE, etc), the flagellar export system (*i.e*., FlhA, FlhB), and other regulatory proteins (*i.e.*, FliA and FlgM). FliA, a sigma 28 factor, is a transcriptional activator of class III flagellar genes, whereas FlgM, an anti-sigma factor, is a transcriptional repressor for class-III operon (44). Similarly, FlhF, FlhG, FleN are essential for defining flagella numbers and the correct placement of flagella in various organisms. For example, in *Vibrio, Bacillus*, and other related species, FlhF and FlhG work in coordination to control flagellar location and number. The deletion of FlhF leads to the mis-localization of flagella, and the FlhG deletion caused hyperflagellated phenotype (32, 45). Likewise, in *P. aeruginosa*, deletion of FleN, an ortholog of FlhG, resulted in hyperflagellation and reduced motility (16). Interestingly, the absence of FlhG in *C. jejuni* (*C. jejuni*), apart from inducing flagellation, also resulted in polar minicell formation, a phenotype found during the deletion of the Min system in *E. coli.* The Min system is absent in *C. jejuni* and FlhG or FleN are absent in *E. coli*. Our experiment showed that the deletion of the Min system in *E. coli* generates both motile cells and polar mini cells (Fig. 1*A*). Incidentally, FlhG, FleN, and MinD, belong to the ParA ATPase family and share a high degree of structural and functional similarities. Although the role of FlhG and FleN are well studied in flagellation and motility, the role of the Min system during flagellation in bacteria is not yet known.

**Figure 8:**
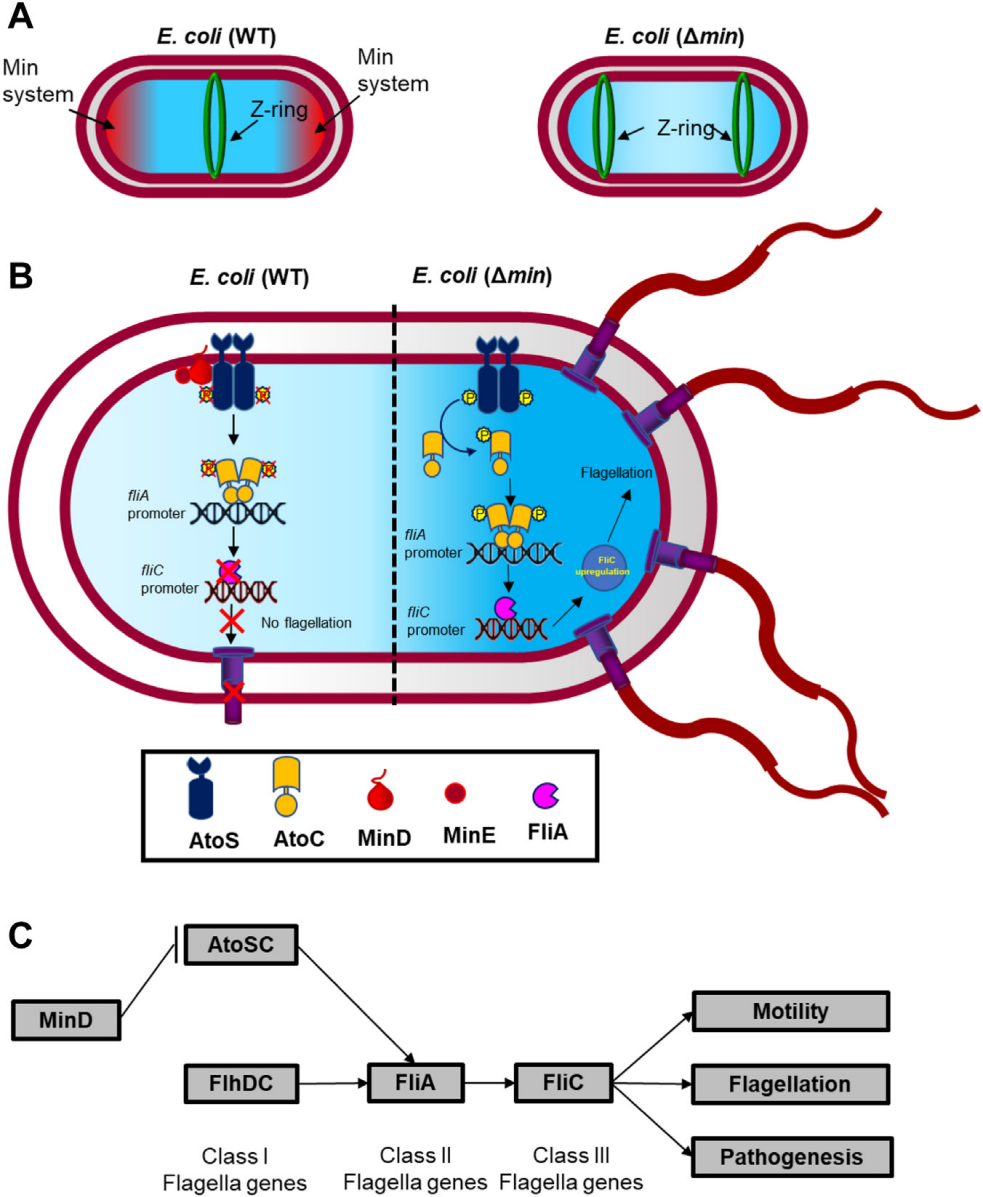
**Mechanism of MinDE mediated flagellation in *Escherichia coli*.** Min deletion *E. coli* shows hyper flagellated morphology. The position of the Z-ring (divisome ring) in *E. coli* (WT), and *E. coli* (Δ*min*) cells is schematically depicted in *A*, which highlights the functions of the Min system in *E. coli* during cell division. *B* and *C*, are showing the suggested mechanism by which MinDE may control the flagellation. Briefly, AtoC of AtoSC complex binds to the *fliA* promoter and controls the *fliA* expression, which in turn controls expression of flagellar genes. MinDE complex interacts with AtoS and hinders binding of AtoC to the *fliA* promoter and thus inhibits flagellation. In the absence of MinDE, AtoSC is free to induce *fliA* expression and thus hyper flagellation occurs.

The increased motility of *E. coli* (Δ *min*) cells made us curious to examine the bacterial morphology and their flagellation pattern. The fluorescence microscopy and TEM imaging showed that the *E. coli* (Δ *min*) cells contain long, dense, and peritrichous flagella (Fig. 2). This result is consistent with the previous findings that the deletion of MinD homologs, that is, FlhG and FleN, etc., leads to multiflagellated phenotypes (16, 45). We wanted to know why such multiflagellated phenotype was found in the absence of the Min system. This phenotype in the *E. coli* (Δ *min*) cells could be due to the higher expression of flagella regulatory genes or the flagellar structural genes. From qRT-PCR analysis, we found that the expression of the flagella regulatory gene *fliA* and the class-III structural genes like *fliC, motA*, and *motB* were increased by several folds in the *E. coli* (Δ *min*) cells (Fig. 3*B*). This result was also supported by the Western blotting analysis, which showed flagellin, a core structural flagellar protein, was highly expressed in these cells (Fig. 3, *C* and *D*). In order to identify if and how the Min system controls the flagellar expression, we need to first understand the flagellar biosynthesis pathway; a concise description is given in Figure 8*C*. Briefly, the master regulator FlhDC (class I flagellar gene) positively controls the expression of the flagellar regulator FliA (class II flagellar gene), which in turn controls the expression of other flagellar proteins (class III flagellar genes). The qRT-PCR data showed that there was an upregulation of most of the flagellar regulators and structural genes (Fig. 3*B*). In addition, Western blot analysis showed that *E. coli* (Δ *min*) cells produce more flagellin than WT cells and that the complementation of the MinDE/MinCDE proteins in *E. coli* (Δ *min*) cells decreased the synthesis of flagellin (Fig. 3, *C* and *D*). This suggested that either MinDE directly interacts with the master regulator FlhDC and controls the expression of downstream genes or controls the expression of flagellar regulator *fliA* expression through binding to its promoter region. Our experiment showed that there is no direct interaction of MinDE with FlhDC (Figs. 6*A* and S3). In order to identify if MinD/E can interact with *fliA* promoter, we performed *in silico* evaluations. The *in silico* analysis showed that like its homologs FleN and FlhG, the DNA binding domain is absent in MinD, suggesting that it may not interact with the *fliA* promoter. Previous studies showed that several MinD homologs (*i.e.*, FlhG/FleN, etc.) negatively control flagellation in different bacteria by interacting with different regulatory proteins (46). For example, FlhG or FleN deletion leads to hyperflagellation morphology similar to *E. coli* (Δ *min*) cells (45). Further, similar to MinD, its homologs FlhG/FleN also lack a DNA binding motif; however, they act as antiactivators for flagellation. Previous studies have reported that FlhG/FleN work with sigma factor 54 activators FlrA/FleQ that directly bind to the promoter region of class-II flagellar gene *fliA* and thus regulate flagellar gene expression (19, 45). So, there is a possibility that MinD has another partner that binds to the promoter region of *fliA* and controls the flagellation in *E. coli*. When FlrA/FleQ sequences were BLAST against the whole *E. coli* K-12 genome, a highly similar protein AtoC was identified. Similar to FlrA and FleQ, AtoC also belongs to the sigma-54–dependent member and is present as a complex AtoSC. This AtoSC complex belongs to the TCS, where AtoS acts as the sensor kinase and AtoC acts as the response regulator. Interestingly, AtoC homologs FlrA and FleQ lack the phosphorylation domain and the genes encoding a sensor kinase upstream of these genes and do not belong to the TCS (47, 48). A study by *Theodorou et al.* has shown that the AtoSC complex controls *E. coli* motility by regulating transcription of both class-I (*flhDC*) and class-II (*fliA*) flagella operons, and AtoSC more effectively enhances the *fliAZY* operons rather than *flhDC* operon (15). The genome-wide promoter analysis of AtoC revealed that the *fliA* promoter contains an AtoC binding site (49). This suggested that to control the flagellation, MinD probably interacts with AtoC. However, our results showed that MinD does not interact with AtoC; instead, it interacted with its complex partner AtoS (Fig. 6*B*). Both MinC and MinE do not interact with either AtoS or AtoC (Fig. S3). AtoS, a sensor histidine kinase, is mostly found in eubacteria that form TCS with AtoC, where AtoC is the response regulator that controls certain gene expressions. In its ATP-bound form AtoS undergoes autophosphorylation and transfers the phosphate group to AtoC, which facilitates dimerization of AtoC. The AtoC dimer binds to the promoter region of target genes and controls their expression (50, 51). As ATP binding and phosphorylation of AtoS is important for controlling the function of AtoC, we speculated that MinD controls AtoSC functions through its binding to the AtoS protein.

Now the question arises; can MinD control flagellar gene expression through AtoSC and how MinD is doing it? Previous studies have shown that MinD homologs FlhG/FleN interact with FlrA/FleQ and repress the expression of flagellar genes (17, 19). In order to identify a similar mechanism, the *fliA* promoter activity was studied in the presence of AtoSC alone or with both AtoSC and MinD (Fig. 6*F*). For this study, native *fliA* promoter was cloned with an eGFP reporter in a promoter-less eGFP vector, where a higher promoter activity will result in higher eGFP expression and will show higher fluorescence. We observed that *fliA* promoter activity was decreased when MinD was expressed in *E. coli* C41 (pET-P4-eGFP/C41) cells, and it increased upon expression of the cyto–AtoSC complex, which further decreased when both cyto-AtoSC and MinD were overexpressed (Fig. 6*F*). These observations confirmed that MinD interacts with the AtoSC complex and negatively regulates *fliA* expression and thus the flagellar expression. As a control, *flhDC* native promoter was used, which did not show any eGFP fluorescence in *E. coli* (Δ*min*) cells, which indicated that MinD neither interacted with FlhDC nor binds to its promoter. This further confirms our hypothesis that FlhDC does not play any role in the MinD-controlled flagellation in *E. coli*.

The AtoSC two-component signal transduction system in *E. coli* plays a crucial role in regulating the expression of genes within the *atoDAEB* operon, which is essential for short-chain fatty acid catabolism. Following the induction by acetoacetate, the AtoS sensor kinase initiates autophosphorylation, leading to the subsequent phosphorylation and activation of the response regulator AtoC. The phosphorylated AtoC binds to the *atoDAEB* promoter and positively regulates its expression. The phosphorylation-deficient mutant of AtoS was unable to activate AtoC. Hence, the functionality of the complex requires phosphorylation of both the proteins. The results of yeast two-hybrid and pull-down assays suggested that MinD interacts with AtoS. Further, the AtoSC complex positively regulates *fliA* promoter expression, and MinDE negatively regulates *fliA* promoter activity. The phosphorylation of AtoC is necessary for controlling *E. coli* motility. So, in order to identify the regulation of MinDE in AtoSC phosphorylation and functions, we studied the *atoDAEB* promoter activity in the presence of MinDE. We hypothesized that if MinDE inhibited AtoS phosphorylation, then the *atoDAEB* promoter activity would be downregulated (Fig. 7*A*). While studying the *atoDAEB* promoter activity in *E. coli* WT and (Δ*min*) cells, we found that MinDE decreased the promoter activity, whereas AtoSC enhanced the *atoDAEB* promoter activity (Fig. 7*B*). These findings indicated that MinDE may inhibit AtoSC phosphorylation.

The Min oscillation is important for the midcell placement of the Z-ring (Fig. 8*A*). Based on our findings, we hypothesize that the AtoSC complex interacts with the *fliA* promoter and positively regulates its expression, which in turn controls the expression of downstream flagellar proteins and flagellar expression. Our findings suggest that in WT *E. coli*, MinD interacts with the AtoS protein of the AtoSC complex and inhibits AtoC’s interaction with the *fliA* promoter through inhibiting AtoS phosphorylation. Due to the lack of this interaction, there is a reduction in *fliA* expression that leads to lower flagellar expression. In the absence of MinD, the AtoSC complex is free to bind to *fliA* promoter and induces *fliA* expression, leading to higher flagellar expression (Fig. 8*B*). Our study suggests that along with the cell division, the Min system is also involved in *E. coli* motility.

## Experimental procedures

### Materials

Hepes, Tris, KCl, NaCl, sodium phosphate, synthetic defined medium -Leu-Trp-His (SD medium), 3-amino-1,2,4-triazole (3-AT), and IPTG were purchased from MP Biomedicals, and LB broth and LB agar were obtained from HiMedia. Anti-flagellin and anti-GroEL antibodies were purchased from Abcam. The remaining chemicals used were of molecular biology grade and were obtained from Sigma-Aldrich.

### Strains and plasmids

*E coli Δmin* (JS964) was a gift from Dr Lutkenhaus lab, and WT lab strain MG1655 (CGSC6300) was used in all experiments (7). The Δ*atoSC* strain was a kind gift from Hirofumi Aiba (Nagoya University) (52). Cloning of *minD*, *atoS*, cyto-*atoS*, and *atoC* in respective plasmid vectors was constructed by amplifying the specific ORF from *E. coli* K-12 genomic DNA. The cloning was confirmed by restriction digestion and DNA sequencing. All the strains and plasmids used in this study are summarized in Tables S1 and S2.

### Protein overexpression and purification

His-MinD and GST-MinD were purified using previously described methods (9). The purified protein was dialyzed against buffer A (50 mM Hepes buffer pH 7.4, 150 mM KCl, 10% glycerol) and stored at −80 °C until further use. *E. coli* BL21 [DE3] cells carrying ORF of the cytoplasmic fraction of AtoS (cyto-AtoS)/BL21 [DE3] cells were grown at 37 °C in Luria–Bertani medium containing 50 μg/ml kanamycin. At *A*_600_ ∼0.5, 1 mM IPTG was added to the cells to induce recombinant protein production (50). Cells were harvested using centrifugation after 4 h postinduction, resuspended in ice-cold lysis buffer [50 mM Hepes pH 7.4, 300 mM KCl, and 1% (v/v) Triton X-100] containing lysozyme (60 μg/ml), protease inhibitor cocktail, and cells were broken using Stansted pressure cell homogenizer. The cell lysate was centrifuged at 10,000*g* for 30 min at 4 °C, and the supernatant was treated with DNase I (6 units/ml) for 10 to 15 min at 4 °C. The lysate was further centrifuged at 10,000*g* for 30 min at 4 °C, and the supernatant was subjected to affinity chromatography using Ni^2+^–NTA agarose column (37). The column was washed with ten column volumes of washing buffer (50 mM Hepes pH 7.4, 300 mM KCl, and 20 mM imidazole). His-tagged proteins were then eluted with 50 mM Hepes (pH 7.4) buffer containing 300 mM KCl and 300 mM imidazole.

### Motility assay

*E. coli* WT (MG1655), *E. coli* (Δ*min*), and *E. coli* (Δ*min*) complemented with different Min components were grown in M-media (LB broth + 0.5% NaCl). From this, 3 μl of culture was spotted on plates containing 0.3% agar in M-media and grown at 37 °C. The motility was observed visually after 12 to 18 h. For live-cell motility imaging, WT *E. coli* (MG1655), *E. coli* (Δ*min*), and Δ*min* cells complemented with *minCDE* were inoculated into the LB broth and grown overnight at 37 °C. From this, 1% of the culture was inoculated and grown in a flat, wide-bottom flask at 37 °C until *A*_600_ reached ∼0.5, where it was added with 1 mM IPTG and grown for 2 h at 60 rpm. From this, 2 μl of culture was placed on the glass slide, and the motility of the cells was observed under the live-cell imaging microscope (Zeiss, cell discoverer) with a 50× water immersion lens.

### Flagella staining and imaging

Flagella were stained with Alexa-488 fluorescent dye as described previously with slight modification (53). Briefly, *E. coli* WT (MG1655), *E. coli* (*Δmin*), and different Min complementation strains were inoculated into the LB broth and grown at 37 °C overnight. From this, 1% of the culture subinoculated into the M-media and allowed to grow at 37 °C for 5 to 6 h at 60 rpm in a flat, wide-bottom flask. Culture (0.5 ml) was pelleted at 1000*g* and resuspended in 1 ml of 0.01 M potassium phosphate buffer, pH 7.4, containing 67 mM NaCl, 0.4 mM EDTA, and 0.002% Tween 20. To 500 μl of the bacterial suspension, 10 μl of Alexa-488 (5 mg/ml) and 50 μM of sodium bicarbonate were added and incubated for 2 h at room temperature. The suspension was washed twice with the above buffer at 500*g* for 5 min. Two microliters sample was taken on a glass slide and observed under a confocal microscope (Leica STED-SP8 microscope). For electron microscopy analysis of flagella, *E. coli* (WT), *E. coli* (*Δmin*), and *E. coli* (*Δmin*) containing min components were grown in M-media, and bacteria were fixed with 2.5% glutaraldehyde for 10 min. Negative staining was performed using the previously described protocol (3). A drop of bacteria culture was applied to a formvar-carbon coated copper grid, negatively stained with 2% uranyl acetate, air dried, and were visualized using a transmission electron microscope (JEOL-TEM JEM-2100 Plus).

### RT-PCR analysis of flagellar genes

*E. coli* cells from the motility area of WT and *Δmin* was swabbed into the motility media and grown at 37 °C. From the exponentially growing cell, total RNA was isolated using a bacterial RNA isolation kit (Qiagen), and complimentary DNA was prepared from 0.2 μg of total RNA using PrimerScript RTase reverse transcriptase (Takara). The resulting DNA was quantified and subjected to qRT-PCR using SYBER Green Master mix (Applied Biosystems). The relative expression of target genes was calculated using the 2^−ΔΔCT^ method by taking 16srRNA as a reference gene (54, 55).

### Immunoblotting

An equal number of bacteria cells *E coli* (WT), *E. coli* (*Δmin*), and *E. coli* (*Δmin*) complemented with different Min components were loaded onto the SDS-PAGE. After electrophoresis separation, the samples were transferred onto a poly(vinylidene fluoride) membrane using a Mini *Trans* Blot apparatus (Bio-Rad) at 90 V for 2 h in Towbin transfer buffer. Blots were blocked using 5% skim milk prepared in Tris-buffered saline with Tween 20 for 1 h. To detect the flagellar filament protein flagellin, the membrane was immune-stained with the rabbit anti-flagellin polyclonal antibody with a dilution of 1:10,000 (Abcam-ab93713), and after washing with Tris-buffered saline with Tween 20, the blot was incubated with anti-rabbit antibody labeled with horseradish peroxidase (anti-rabbit-HRP-A6154) (1:10,000). The anti-GroEL monoclonal antibody was taken as loading control (Abcam-ab82592), and anti-mouse antibody (1;10,000 dilution) was used as the secondary antibody. The expression of flagellin was quantified densitometrically using ImageJ software (https://imagej.net/ij/).

### Yeast two-hybrid assay

Yeast two-hybrid analysis was performed using the protocol mentioned earlier, where the yeast strain HFY7c containing plasmids pGAD424 (Clontech) with the GAL4 activating domain (GAL4-AD) and PGBT9 (Clontech) with the GAL4 DNA-binding domain (GAL4-BD) were used (56). For the yeast two-hybrid analysis, HIS3 was used as a nutritional reporter system. Briefly, yeast strain HFY7c was grown at 30 °C in YPD broth until midlog phase (*A*_600_ = 0.5–0.8). Once it reached the midlog phase, competent cells were prepared, and various combinations of GAL4-AD and GAL4-BD containing MinC, MinD, AtoC, AtoS FlhD, FlhC, FliA, and FliC were cotransformed into the competent cells. The positive transformants were screened on synthetic defined medium (SD medium) without leucine and tryptophan. To verify interaction, cotransformants were spotted on -Leu-Trp-His selection media plates without and with various concentrations of 3-AT and grown at 30 °C for 3 to 4 days. Yeast transformants exhibiting growth on plates lacking histidine and the presence of 3-AT suggest a positive protein–protein interaction. Each interaction was investigated in biological triplicate experiments.

### GST-MinD/His-cyto-AtoS pull-down assay

GST-MinD (5 μM) was incubated with His-cyto-AtoS (5 μM) in buffer-B (50 mM Hepes pH 7.4, 150 mM KCl) at room temperature for 30 min. HisPure cobalt resin was added to the reaction mixture and incubated for 1 h at 4 °C. His-cyto-AtoS (5 μM) or GST-MinD (5 μM) alone were used as controls. The whole reaction mixture was transferred to a spin column, washed three times with buffer B containing 20 mM imidazole and eluted with buffer containing 50 mM Hepes pH 7.4, 150 mM KCl, and 300 mM imidazole. The GST-tagged MinD fraction eluted along with His-cyto-AtoS was verified on a 12% SDS-PAGE and also subjected to Western blot analysis. Anti-GST (Invitrogen: 136700), anti-His primary antibodies (Sigma), and anti-mouse-horseradish peroxidase–conjugated secondary antibodies (Sigma) were used for the detection of MinD and cyto-AtoS. All antibodies were used at a 1:10,000 dilution.

### Promoter activity assay

To study the native promoter activity of *flhDC* and *fliA* through the eGFP reporter assay, we first cloned the *egfp* gene in the pET22b vector and removed the T7 promoter by digestion with BglII and NdeI restriction enzymes. Next, we identified the promoter regions of *flhDC* and *fliA* from the *E. coli* K-12 genome, and different truncated parts of the promoter were PCR amplified using specific primers. These promoter regions were cloned into the upstream eGFP of the promoter-less pET22b-eGFP vector at BglII and NdeI sites. The clone was confirmed by restriction digestion and sequencing. To study the promoter activity, we transferred the plasmids containing various truncated *flhDC* promoter (pET-P1-eGFP, pET-P2-eGFP, pET-P3-eGFP) and pET-P4-eGFP plasmid (containing *fliA* promoter) into *E. coli* (WT) and *E. coli* (Δ*min*) cells. Further, to verify the roles of MinD and AtoSC on *fliA* promoter activity, we transferred pACYCDuet-1-*minD* and pCDFDuet-1-cyto*-atoSC* to the pET-P4-eGFP/C41strain. The strains containing respected plasmid were grown at 37 °C and at *A*_600_ ∼0.5 induced with 1 mM IPTG for 3 h. An equal number of cells were loaded on an SDS-PAGE, and Western blot was performed using an anti-GFP mAb (Invitrogen: 332600). Anti-GroEL antibody (Abcam: ab82592) was used as a loading control. Anti-mouse antibody was used as secondary antibody to stain the blot. All the antibodies used in this experiment were 1:10,000 dilutions.

### Reporter assay for *fliA* promoter activity

For this assay, *fliA* native promoter from *E. coli* was cloned in pCDFDuet-1- eGFP plasmid and both the T7 promoter was removed. This plasmid was transferred into *E. coli* (WT), *E. coli* (Δ *min*), and *E. coli* (Δ *min*) with Min complementation strains, streaked on a LB agar plate containing respective antibiotics, 0.1 mM IPTG and grown at 37 °C for 12 to 16 h. Images were taken on the Bio-Rad Chemidoc imaging system. To measure the eGFP intensity, cells were grown until *A*_600_ ∼ 0.5 at 37 °C and induced with 1 mM IPTG for 3 h. Next, the cells were washed with 0.85% NaCl, and an equal number of cells were taken for fluorimeter to measure the eGFP fluorescence intensity.

### *atoDAEB* promoter activity

In alignment with the above-describe procedures, the *atoDAEB* promoter was cloned into the eGFP-pET22b vector following the elimination of the T7 promoter. Subsequently, the resulting patoD1-eGFP-pET22b vector was introduced into *E. coli* (WT) and into *E. coli* (Δ *min*), along with MinDE and AtoSC. The cells were grown at 37 ºC in the presence of 10 mM acetoacetate and respective antibiotics. At *A*600 ∼0.5, the cells were induced with 1 mM IPTG for 2 h. Equal numbers of cells were loaded onto an SDS-PAGE, and a Western blot was performed utilizing a mouse anti-GFP mAb (Invitrogen: 332600). Anti-GroEL mAb (Abcam: ab90522) was used as a loading control

## Data availability

All data are contained within the manuscript.

## Supporting information

This article contains supporting information.

## Conflict of interest

The authors declare that they have no conflicts of interest with the contents of this article.
