# Supplementary material for: The bacterial division protein MinDE has an independent function in flagellation
Source: J Biol Chem. 2024 Feb 23;300(4):107117. doi: 10.1016/j.jbc.2024.107117 (PMC10963238; doi:10.1016/j.jbc.2024.107117)
Supplement: Supporting Information [file mmc4.docx]

**Supporting Information:**

**Supplementary Figures:**

**
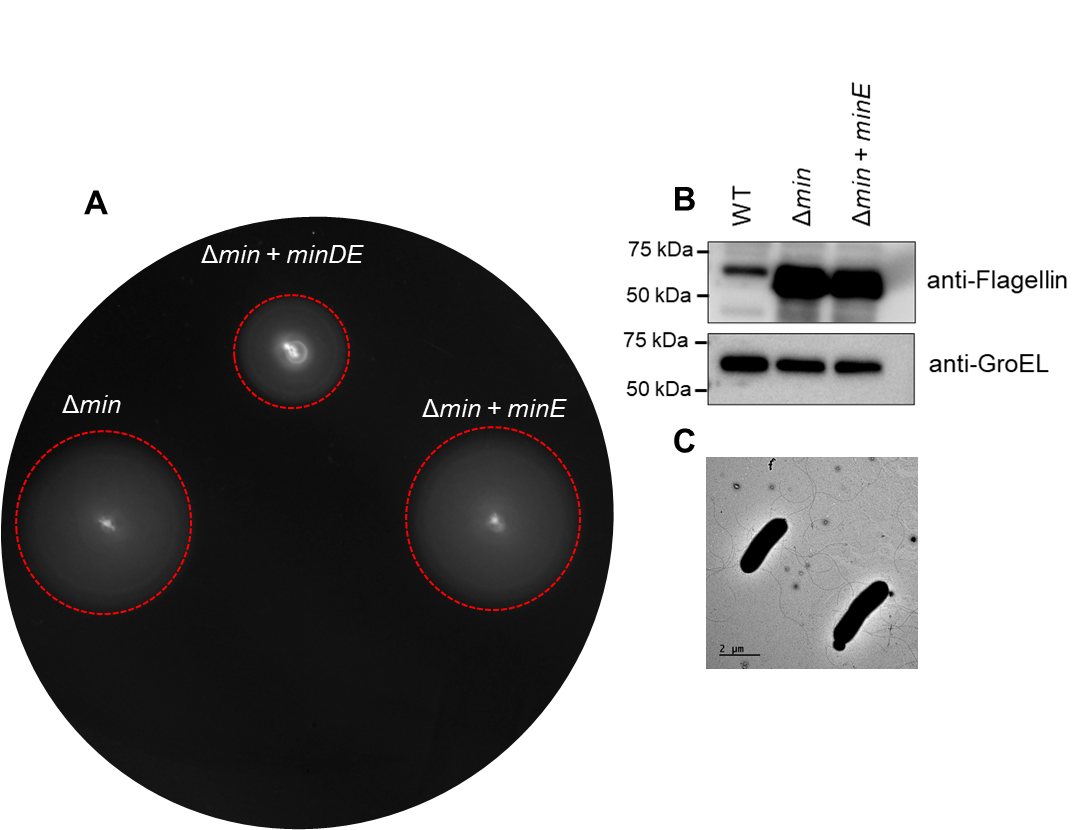
Figure S1**. ***Effect of MinE in E. coli (Δ min) motility.***  ***A*,** *shows E. coli* JS964 (Δ*min*), *minDE* and *minE* complemented *E. coli (*Δ*min)* cells were grown overnight, and 3 µl of culture from each strain was applied to 0.3% soft-agar plates containing ampicillin (100 μg/ml) and IPTG (0.1 mM). The plates were incubated at 37 °C for 12-18 hours, and the bacterial motility was observed. The western blot for flagellin expression of wild type, Δ*min*, *and minE* complemented strain is shown on ***B***. Transmission electron micrographs showing the presence of flagella in *minE* complemented Δ*min* cells are shown in ***C*** (scale bar = 2 µm).

**Figure S2.** ***Schematic representation showing the details of the construction of plasmid for eGFP reporter assays.*** Here *egfp* gene sequence was amplified and cloned at NdeI and HindIII sites in the pET22b vector. Then the T7 promoter was replaced with *fliA* or *flhDC* promoter at BglII and NdeI sites making the final pET-P1-eGFP, pET-P2-eGFP, pET-P3-eGFP and pET-P4-eGFP vector (P1 = *flhDC* 467 bp, P2 = *flhDC* 314 bp, P3 = *flhDC* 398 bp, P4 = *fliA* 350 bp).

**
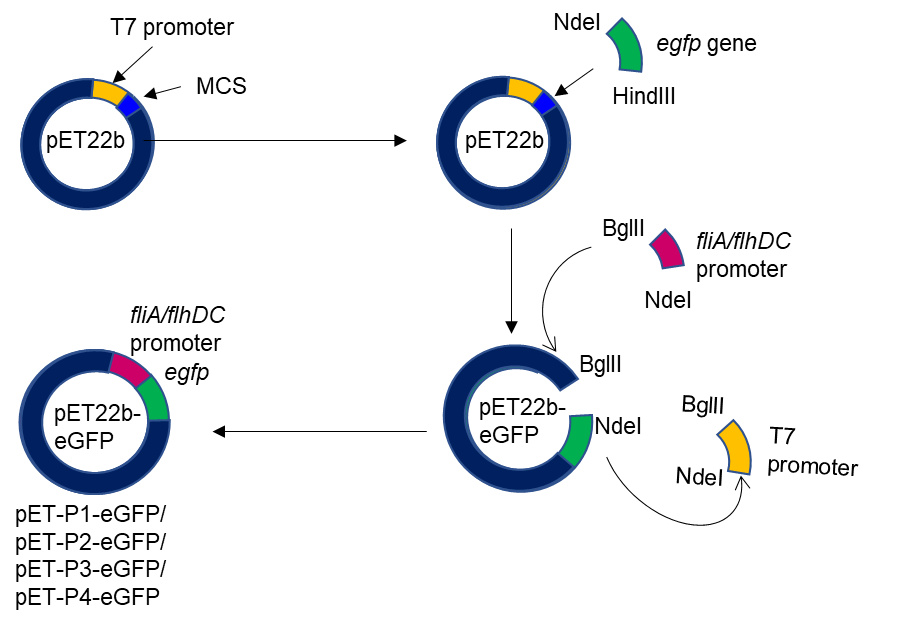
**

**Figure S3: *Yeast two-hybrid assay.*** The figure shows the interaction of MinC and MinE with the flagella regulator and AtoSC two-component system.  ***A*** show the interaction of MinC with flagella regulator FlhDC, FliA, and FliC, and ***B*,** shows the interaction of MinE with FlhDC, FliA, and FliC***.* C**, showing the interaction of MinC with AtoSC. ***D***, showing the interaction of MinE with AtoS and AtoC.

**
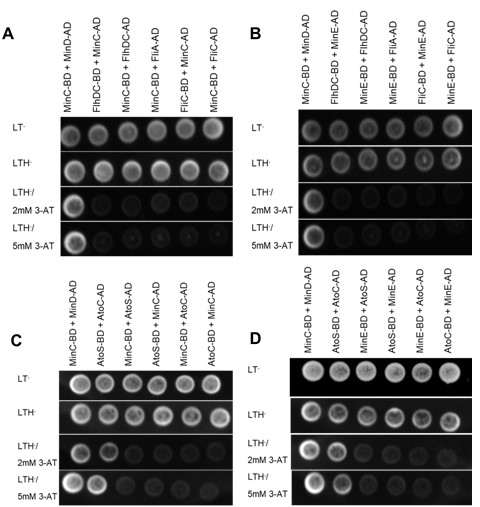
**

**Supplementary Table ST1:** List of strains used in the study.

| Sl No | Strains Name | Stock No. | Source/ Reference |
| --- | --- | --- | --- |
| 1 | JS964 (Δ*min*) | TKB-272 | Hu, Z., and Lutkenhaus,J. (7) |
| 2 | BW28878 (Δ*atoSC*) | TKB-634 | Oshima, T., *et al* (52) |
| 3 | pTrc99a + MG1655 | TKB-440 | In this study |
| 4 | pTrc99a+ Δ*min* | TKB-441 | In this study |
| 5 | *minC* + Δ*min* | TKB-275 | In this study |
| 6 | *minD*+ Δ*min* | TKB-273 | In this study |
| 7 | *minE*+ Δ*min* | TKB-546 | In this study |
| 8 | *minDE* + Δ*min* | TKB-276 | In this study |
| 9 | *minCDE* + Δ*min* | TKB-274 | In this study |
| 10 | Promoterless/ pET22b-eGFP /DH5α | TKB-478 | In this study |
| 11 | pET-P1-eGFP/MG1655 (P1 WT) | TKB-474 | In this study |
| 12 | pET-P1-eGFP/ Δ*min* (P1 Δ*min*) | TKB-472 | In this study |
| 13 | pET-P2-eGFP /MG1655 (P2 WT) | TKB-473 | In this study |
| 14 | pET-P2-eGFP / Δ*min* (P2 Δ*min*) | TKB-470 | In this study |
| 15 | pET-P3-eGFP MG1655 (P3 WT) | TKB-469 | In this study |
| 16 | pET-P3-eGFP / Δ*min* (P3 Δ*min*) | TKB-467 | In this study |
| 17 | pET-P4-eGFP/MG1655 (P4 WT) | TKB-468 | In this study |
| 18 | pET-P4-eGFP/ Δ*min* (P4 Δ*min*) | TKB-475 | In this study |
| 19 | pCDFDuet-1- *fliAp-*eGFP / MG1655 | TKB-523 | In this study |
| 20 | pCDFDuet-1- *fliAp-*eGFP / Δ*min* | TKB-514 | In this study |
| 21 | pCDFDuet-1- *fliAp-*eGFP /pTrc99a-*minC* + Δ*min* | TKB-527 | In this study |
| 22 | pCDFDuet-1- *fliAp-*eGFP /pTrc99a -*minD*+ Δ*min* | TKB-526 | In this study |
| 24 | pCDFDuet-1- *fliAp-*eGFP /pTrc99a – *minCDE* + Δ*min* | TKB-552 | In this study |
| 25 | pCDFDuet-1- *fliAp-*eGFP /pTrc99a – *minD*E + MG1655 | TKB-521 | In this study |

**Supplementary Table ST2:** List of plasmids used in the study

| Sl No | Plasmid name | Description | Reference |
| --- | --- | --- | --- |
| 1 | pGBT9-*minD* | *minD* cloned in pGBT9 | In this study |
| 2 | pGAD424- *minD* | *minD* cloned in pGAD424 | In this study |
| 3 | pGBT9-*minC* | *minC* cloned in pGBT9 | In this study |
| 4 | pGAD424- *minC* | *minC* cloned in pGAD424 | In this study |
| 5 | pGBT9-*minE* | *minE* cloned in pGBT9 | In this study |
| 6 | pGAD424- *minE* | *minE* cloned in pGAD424 | In this study |
| 7 | pGBT9-*atoC* | *atoC* cloned in pGBT9 | In this study |
| 8 | pGAD424- *atoC* | *atoC* cloned in pGAD424 | In this study |
| ~~9~~ | pGBT9-*atoS* | *atoS* cloned in pGBT9 | In this study |
| 10 | pGAD424- *atoS* | *atoS* cloned in pGAD424 | In this study |
| 11 | pGBT9-*flhD* | *flhD* cloned in pGBT9 | In this study |
| 12 | pGBT9-*flhC* | *flhC* clone in pGBT9 | In this study |
| 13 | pGBT9-*flhDC* | *flhDC* clone in pGBT9 | In this study |
| 14 | pGAD424-*flhDC* | *flhDC* clone in pGAD424 | In this study |
| 15 | pGBT9-*fliA* | *fliA* clone in pGBT9 | In this study |
| 16 | pGAD424-*fliA* | f*liA* clone in pGAD424 | In this study |
| 17 | pGBT9-*fliC* | *fliC* clone in pGBT9 | In this study |
| 18 | pGAD424-*fliC* | *fliC* clone in pGAD424 | In this study |
| 19 | pET28a- cyto-*atoS* | cyto-*atoS* clone in pET28a for expression | In this study |
| 20 | pGEX6P1-*minD* | *minD* cloned in pGEX6P1 vector for protein expression | Taviti, A. C., and Beuria, T. K. (9) |
| 21 | *minC* | *minC* cloned in pTrc99a | In this study |
| 22 | *minD* | *minD* cloned in pTrc99a | In this study |
| 23 | *minE* | *minE* cloned in pTrc99a | In this study |
| 24 | *minDE* | *minDE* cloned in pTrc99a | In this study |
| 25 | *minCDE* | *minCDE* cloned in pTrc99a | In this study |
| 26 | pET22b-eGFP | *egfp* gene cloned in pET22b | In this study |
| 27 | pET-P1-eGFP (P1) | *flhDC* promoter (467bp) cloned in pET22b-eGFP plasmid after removing the T7 promoter | In this study |
| 28 | pET-P2-eGFP (P2) | *flhDC* promoter (315bp) cloned in pET22b-eGFP plasmid after removing the T7 promoter | In this study |
| 29 | pET-P3-eGFP (P3) | *flhDC* promoter (382bp) cloned in pET22b-eGFP plasmid after removing the T7 promoter | In this study |
| 30 | pET-P4-eGFP (P4) | *fliA* promoter cloned in pET22b-eGFP plasmid after removing the T7 promoter | In this study |
| 31 | Promoter- less pET22b-eGFP | T7 promoter was removed from pET22b-eGFP plasmid to generate the promoterless- pET22b-eGFP vector | In this study |
| 32 | pCDFDuet-1- *fliAp-*eGFP | Native *fliA* promoter cloned in pET22b-eGFP plasmid was digested and ligated it in pCDFDuet-1 vector after removing the T7 promoter for promoter assay | In this study |
| 33 | pACYCDuet-1- *tac* | The T7 promoter of pACYCDuet-1 was replaced with *tac*-promoter of pFLAG-MAC | In this study |
| 34 | pCDFDuet-1- *atoSC* | For dual expression of AtoSC it cloned under the T7 promoter of pCDFDuet-1 | In this study |
| 35 | pACYCDuet-1- *tac* -*minD* | For expression of MinD | In this study |
| 36 | pET22b-atoDAEBp-eGFP (patoD1-eGFP-pET22b) | Native *atoDAEB* promoter cloned in pET22b-eGFP after removing the T7 promoter for promoter assay | In this study |
| 37 | pACYCDuet-1- *tac* -*minDE* | For expression of MinDE | In this study |
| 38 | pACYCDuet-1- *tac* -*atoSC* | For expression of full-length AtoSC proteins | In this study |
